# Potential SLA Hp-4.0 haplotype-restricted CTL epitopes identified from the membrane protein of PRRSV induce cell immune responses

**DOI:** 10.3389/fmicb.2024.1404558

**Published:** 2024-05-22

**Authors:** Tingyu Luo, Chang Xin, Hongyi Liu, Changwen Li, Hongyan Chen, Changyou Xia, Caixia Gao

**Affiliations:** State Key Laboratory for Animal Disease Control and Prevention, Heilongjiang Provincial Key Laboratory of Laboratory Animal and Comparative Medicine, National Poultry Laboratory Animal Resource Center, Harbin Veterinary Research Institute, Chinese Academy of Agricultural Sciences (CAAS), Harbin, China

**Keywords:** PRRSV, membrane protein, Hp-4.0 haplotype, SLA restriction, CTL epitope

## Abstract

Swine leukocyte antigen (SLA) class I molecule-restricted T-cell epitopes, which induce cytotoxic T lymphocyte (CTL) responses, play a critical role in the clearance of porcine reproductive and respiratory syndrome virus (PRRSV) and the development of efficient protective vaccines. The SLA-1*04:01:01, SLA-2*04:01, and SLA-3*04:01 alleles, assigned the Hp-4.0 haplotype, are highly prevalent and usually present in all pig breeds. However, the SLA Hp-4.0 haplotype-restricted CTL epitopes in the structural membrane (M) protein of PRRSV are still unknown. In this study, we predicted 27 possible 9-mer epitope peptides in M protein with high binding scores for SLA-1*04:01:01 using CTL epitope prediction tools. In total, 45 SLA class I complexes, comprising the predicted peptide, extracellular region of the SLA-I molecules, and β2-microglobulin, were constructed *in vitro* to detect the specific binding of these peptides to SLA-1*04:01:01 (27 complexes), SLA-2*04:01 (9 complexes), and SLA-3*04:01 (9 complexes), respectively. Our results showed that the M27 (T_91_WKFITSRC), M39 (N_130_HAFVVRRP), and M49 (G_158_RKAVKQGV) peptides bind specifically to SLA-1*04:01:01, SLA-2*04:01, and SLA-3*04:01, respectively. Subsequently, using peripheral blood mononuclear cells (PBMCs) isolated from the homozygous Hp-4.0 and Hp-26.0 haplotype piglets vaccinated with commercial PRRSV HuN4-F112 strain, we determined the capacities of these 27 potential peptides to stimulate their proliferation with a Cell Counting Kit-8 and their secretion and expression of interferon gamma (IFN-γ) with an ELISpot assay and real-time qPCR, respectively. The immunological activities of M27, M39, and M49 were therefore confirmed when they efficiently induced PBMC proliferation and IFN-γ secretion in PBMCs from piglets with the prevalent SLA Hp-4.0 haplotype. The amino acid sequence alignment revealed that M27, M39, and M49 are highly conserved among 248 genotype II PRRSV strains collected between 1998 and 2019. These findings contribute to the understanding of the mechanisms of cell-mediated immune responses to PRRSV. Our study also provides a novel strategy for identifying and confirming potential SLA haplotype-restricted CTL epitopes that could be used to develop novel peptide-based vaccines against swine diseases.

## 1 Introduction

Porcine reproductive and respiratory syndrome (PRRS) is an economically important viral disease in the pig industry worldwide and has caused huge economic losses in Chinese pig production (Zhou and Yang, 2010). The continuous evolution of the PRRS virus (PRRSV) in genetically and antigenically diverse field strains has hampered the development of safe and broadly protective vaccines (Nilubol et al., 2004). The commercially modified live-attenuated vaccines currently available are not broadly cross-protective against all heterologous PRRSV strains and may also be related to many new PRRSV strains because recombination is involved in the evolution of these viruses (Gao J. C. et al., 2017). Therefore, there are considerable challenges and specific requirements in the development of novel vaccines to prevent PRRS, including cytotoxic T lymphocyte (CTL) epitope vaccines. A number of studies have shown that the cell-mediated immune response to CTL epitopes conserved across influenza A virus strains are protective against influenza (Belz et al., 2002; Lee et al., 2008; Price et al., 2009). Previous research has also suggested that cell-mediated immunity plays a very important role in the clearance of PRRSV and in controlling viral infection (Murtaugh et al., 2002). However, we still lack a systematic and comprehensive understanding of the immune mechanisms of PRRS.

The cell-mediated immune response depends on the recognition by T-cell receptors of peptides bound to major histocompatibility complex (MHC) class I molecules which are presented on the surfaces of cells. Identification of epitope peptide binding to MHC class I molecules represents a critical step in this process. Swine MHC, referred to as swine leukocyte antigen (SLA), has been mapped to pig chromosome 7 and comprises highly polymorphic classical class I, class II, and conservative class III gene clusters (Renard et al., 2006). The SLA class I gene cluster contains three constitutively expressed classical genes, namely, *SLA-1*, *SLA-2*, and *SLA-3*, which are highly polymorphic (Hammer et al., 2020). A total of 229 classical SLA class I alleles (89 *SLA-1*, 97 *SLA-2*, and 43 *SLA-3*) are designated by the SLA Nomenclature Committee of the International Society for Animal Genetics (ISAG) in the Immuno Polymorphism Database (IPD)-MHC SLA sequence database^1^ [Release 3.9.0.1 (2022-07) build 209] (Maccari et al., 2017). As of July 2019, 73 independent class I alleles have been assigned haplotypes (Hammer et al., 2020). Among these haplotypes, SLA class I haplotype Hp-4.0 (SLA-1*04:01:01-SLA-3*04:01-SLA-2*04:01) is commonly present in commercial pigs, native pigs, and swine cell lines (Gao et al., 2017b). The three-dimensional structure of the peptide-SLA-1*040101 molecule was also the first to be determined and displayed the peptide presentation characteristics of the SLA-1*04:01:01 molecule in swine (Zhang et al., 2011). Therefore, given the strict SLA class I molecule restriction of CTL epitopes, the identification of prevalent SLA Hp-4.0 haplotype-restricted CTL epitopes could facilitate the rational development and modification of epitope-based vaccines to protect the majority of the pig population carrying the highly prevalent SLA haplotypes.

It has been reported that the PRRSV non-glycosylated membrane (M) protein induces a virus-specific T-cell response and is involved in cell-mediated immunity (Jeong et al., 2010). However, SLA Hp-4.0 haplotype-restricted CTL epitopes in M protein have not yet been identified. In this study, we used a rational approach to identify potential SLA Hp-4.0 haplotype-restricted CTL epitopes that are highly conserved among genotype II PRRSV strains. First, we predicted and evaluated potential 9-mer SLA-1*040101-restricted epitope peptides in the M protein using CTL epitope prediction tools. The candidate epitope peptides were synthesized to construct the peptide-SLA-β2m complexes *in vitro*. The specific binding of these peptides to SLA-1*04:01:01, SLA-2*04:01, and SLA-3*04:01 was analyzed using an ELISA-based method to define the SLA restrictions. Next, peripheral blood mononuclear cells (PBMCs) collected from homozygous Hp-4.0 and Hp-26.0 haplotype piglets vaccinated with a commercial PRRSV HuN4-F112 strain were stimulated with the predicted peptides. PBMC proliferation was detected with the Cell Counting Kit-8 (CCK-8); interferon gamma (IFN-γ) secretion and expression in the PBMCs were measured with an ELISpot assay and real-time quantitative PCR (qPCR), respectively. Finally, we compared the amino acid sequence conservation of the identified potential SLA Hp-4.0 haplotype-restricted CTL epitopes in the M proteins of 248 genotype II PRRSV strains collected between 1998 and 2019. Our results provide a framework for the further study of cellular immune mechanisms and the development of effective polypeptide vaccines.

## 2 Materials and methods

### 2.1 Viruses and animals

The commercial PRRSV HuN4-F112 strain was used in this study. In total, 12 1-month-old specific pathogen-free (SPF) Landrace piglets with homozygous SLA haplotypes, including six Hp-4.0 (SLA-1*04:01:01-SLA-3*04:01-SLA-2*04:01) pigs and six Hp-26.0 (SLA-1*08:11-SLA-3*05:02-SLA-2*10:06) pigs from the breeding stock maintained in the isolation environment at the Harbin Veterinary Research Institute (HVRI) of the Chinese Academy of Agricultural Sciences (CAAS) (Harbin, China), were screened as previously described (Gao et al., 2017b, 2019). SPF Landrace pigs are free of 27 major swine diseases and the corresponding antibodies. The animal experiment was conducted strictly following the guidelines of the Animal Ethics Committee of HVRI, CAAS, China. The approval number of the Animal Ethics Committee of Heilongjiang Province, China, is no. 180428-01.

### 2.2 Computational prediction and synthesis of SLA-1*040101-restricted CTL epitopes in M protein

The potential 9-mer SLA-1*040101-restricted epitopes of PRRSV M protein were predicted and evaluated based on the presentation by SLA-1*04:01:01 with the CTL epitope prediction tools, ICES^2^ (Liao et al., 2013), IEDB Analysis Resource,^3^ and NetMHCpan^4^ (Hoof et al., 2009). The 27 peptides with higher binding scores were synthesized and purified to a purity of >95% with high-performance liquid chromatography (Table 1). Each of the 27 epitope peptides was then dissolved in ddH_2_O or DMSO to a stock concentration of 500 μg/mL.

**Table 1:** Predicted peptides chemically synthesized and screened to identify SLA-1*040101-restricted CTL epitopes of PRRSV M protein.

Peptide no.	Amino acid sequence	Position of residues	IEDB rank	NetMHCpan rank
M1	STAPQKVLL	12–20	40	1.8
M2	VLLAFSITY	18–26	40	1.2
M3	LKVSRGRLL	34–42	96	61
M4	GLLHLLIFL	43–51	42	
M5	FGYMTFVHF	57–65	27	
M6	HFESTNRVA	64–72	22	
M7	LTMGAVVAL	73–81	56	2.3
M8	YSAIETWKF	86–94	65	0.74
M9	KFITSRCRL	93–101	61	12
M10	TSRCRLCLL	96–104	100	
M13	DFCNDSTAP	7–15	55	94
M14	DSTAPQKVL	11–19	94	
M15	PQKVLLAFS	15–23		95
M16	QKVLLAFSI	16–24	93	65
M17	VMIYALKVS	29–37	88	
M22	LHLLIFLNC	45–53	38	98
M23	TNRVALTMG	68–76	100	82
M26	IETWKFITS	89–97	73	75
M27	TWKFITSRC	91–99	82	85
M31	LGRKYILAP	104–112	98	83
M33	VESAAGFHP	116–124	75	73
M34	GFHPIAAND	121–129	33	81
M39	NHAFVVRRP	130–138	61	92
M44	NGTLVPGLK	144–152	82	
M45	PGLKSLVLG	149–157	45	94
M46	LKSLVLGGR	151–159	89	90
M49	GRKAVKQGV	158–166	93	91

### 2.3 Specific binding of predicted epitope peptides to SLA-1*04:01:01 molecule *in vitro*

The specific binding of SLA-1*04:01:01 to the potential epitope peptides of the PRRSV M protein was analyzed with a relatively simple and rapid *in vitro* refolding ELISA-based method, as previously described (Gao et al., 2017a), with protein of NS4A-SLA-1*1301-β2m serving as positive control (PC) and NS4A-SLA-1*040101-β2m serving as negative control (NC). In brief, the double-stranded protein constructs, pET-SLA-1*040101-β2m and pET-epitope-SLA-1*040101-β2m (“epitope” indicates the predicted epitope peptide), consisting of the predicted epitope peptide linked to SLA-1*04:01:01 and β2m, were constructed from single-stranded nucleotide sequences encoding the epitope peptide and a glycine-rich linker (Supplementary Table S1) with the splicing by overlap extension (SOE)-PCR method (Supplementary Figure S1). The recombinant proteins were expressed in a prokaryotic expression system, purified, and refolded. Nickel-coated plates were coated with the refolded proteins at room temperature overnight and washed three times. Monoclonal antibody PT85A was then incubated for 1 h at 37°C. PT85A can recognize all SLA class I molecules and the conformational epitope, which requires the presence of the “correct” peptide and the “correct” SLA class I molecule sequence (Gao et al., 2017a). Following washing, a horseradish peroxidase (HRP)-conjugated goat anti-mouse IgG (H + L) antibody was incubated for 1 h at 37°C, followed by washing three times. Tetramethylbenzidine (TMB) was added, and the reaction was stopped by adding 2 M H_2_SO_4_. The absorbance value was measured immediately at 450 nm. The specific binding of candidate epitope peptides to SLA-1*04:01:01 molecule was determined by a relative optical density (OD) value of ≥2.0, which was calculated as OD (pET-epitope-SLA-1*040101-β2m)/OD (pET-SLA-1*040101-β2m). The same procedures were performed for positive and negative controls. Each sample was assayed in triplicate to determine the mean value.

### 2.4 Specific binding of SLA-1*04:01:01-binding peptides to SLA-2*04:01 and SLA-3*04:01 molecules *in vitro*

The specific peptides bound to SLA-1*04:01:01 were first screened according to the above results. Double-stranded nucleotides encoding these epitope peptides were then constructed and ligated individually to pET-SLA-2*0401-β2m and pET-SLA-3*0401-β2m. The recombinant proteins were expressed as inclusion bodies and refolded. The specific binding of these peptides to SLA-2*04:01 and SLA-3*04:01 was analyzed with the ELISA-based method described above.

### 2.5 Vaccination of SPF landrace piglets with homozygous SLA haplotypes

Six homozygous SLA Hp-4.0 haplotype Landrace piglets and six homozygous SLA Hp-26.0 Landrace piglets were screened for use in the vaccination experiment. The extraction of the total RNA from PBMCs isolated from piglets, synthesis of cDNA, and reverse transcription-PCR amplification of the complete coding sequences of three SLA loci (*SLA-1*, *SLA-2*, and *SLA-3*) were carried out as described previously (Gao et al., 2017b). All PCR products were purified, and nucleotide sequences were determined by direct sequencing. Subsequently, the piglets of each homozygous haplotype were randomly divided into two groups: vaccinated group (*n* = 3) and unvaccinated negative control group (*n* = 3). The piglets of the vaccinated group were injected intramuscularly with 3 × 10^4.5^ 50% tissue culture infectious dose (TCID_50_) of the PRRSV HuN4-F112 strain. The piglets in the negative control groups were injected with an equivalent volume of dilutions. At 17 days post vaccination (DPV), blood samples were collected from all the piglets for the isolation of PBMCs. Serum samples collected from all the piglets at 0, 1, 7, 14, and 19 DPV were assayed for anti-PRRSV antibodies with the PRRSV antibody test kit (INGENASA, Madrid, Spain).

### 2.6 Detection of PBMC proliferation with CCK-8

The proliferation of PBMCs was detected with the CCK-8 (Beyotime Institute of Biotech, China), according to the manufacturer’s instructions. Initially, all predicted epitope peptides were distributed into six groups (Table 2). Fresh PBMCs from each SLA haplotype piglet were cultured in RPMI-1640 medium supplemented with 10% fetal bovine serum in 96-well plates at 10^5^ cells per well. The cultured cells were stimulated with the six peptide groups for 72 h. PBMCs incubated with ddH_2_O or DMSO were used as the negative control, and cells treated with 10 μg/mL phytohemagglutinin (PHA) were used as the positive control. Each treatment contains triplicate wells. After treatment, 10 μL of CCK-8 solution was added to each well, and the cells were incubated for a further 5 h. The OD value of each well was measured at 450 nm. The stimulation index for the vaccinated group or unvaccinated negative control group was calculated as the ratio of the average OD of the peptide-stimulated cells to the average OD of the unstimulated cells for each SLA haplotype. Based on the results of the first round of screening, some peptide groups were selected for further analysis. The stimulation index was further evaluated for individual predicted epitope peptides in the selected groups to stimulate PBMCs.

**Table 2:** Groups of all predicted epitope peptides.

Group	Predicted epitope peptide
Group 1	M1, M2, M6, M8, M9
Group 2	M3, M4, M5, M7, M10
Group 3	M13, M14, M15, M26, M31
Group 4	M16, M17, M22, M23, M27
Group 5	M39, M44, M45, M46, M49
Group 6	M33, M34

### 2.7 Measurement of IFN-γ response with ELISpot assay

Peptide-specific IFN-γ-secreting cells were analyzed with the porcine IFN-γ ELISpot Kit (R&D Systems, United States), according to the manufacturer’s instructions. All tests were performed in triplicate. Six peptide groups (Table 2) were first screened with this assay, and the individual peptides in the selected groups were further evaluated based on the initial results. To perform the assay, 96-well plates pre-coated with anti-IFN-γ antibody were seeded with PBMCs isolated from the vaccinated group piglets or the unvaccinated negative control group piglets at a density of 5 × 10^5^ cells per 100 μL of medium per well. The pooled or individual peptides (10 μg/mL) were then added to the wells. PBMCs incubated with 10 μg/mL PHA were used as the positive control and those incubated with ddH_2_O or DMSO as the negative control. Wells seeded with medium alone were used as the background control. After overnight incubation at 37°C, the cells were discarded, and the plates were treated with a biotinylated anti-IFN-γ detection antibody and streptavidin-alkaline phosphatase. An alkaline phosphatase substrate solution (BCIP/NBT Chromogen) was then added, and the reaction was stopped by washing the wells extensively with tap water until distinct spots emerged. The number of spots was counted with the ELISPOT Image Analyzer and software (Sage Creation Science, Beijing). The spots forming cells (SFC) were defined as the average number of spots in triplicate PBMC samples stimulated with peptide minus the average number of negative spots in triplicate PBMC samples. The data were expressed as the average number of SFC per one million PBMCs (SFC/10^6^ PBMCs).

### 2.8 Detection of IFN-γ mRNA expression with real-time qPCR

PBMCs (2.5 × 10^5^) from the vaccinated or unvaccinated negative control Hp-4.0 haplotype piglets were incubated with the individual peptides in the selected groups. PHA was used for the positive stimulation control and ddH_2_O or DMSO was used for the negative stimulation control. PBMCs were harvested after incubation for 18 h at 37°C. IFN-γ mRNA expression was detected with real-time qPCR according to the method described by Lowe et al. (2014). qPCR was performed with SYBR^®^ Premix Ex Taq^™^ (TaKaRa, Dalian, China), according to the manufacturer’s protocol. All samples were tested in triplicate. The specific primers for porcine IFN-γ were as follows: forward (5′-GCT CTG GGA AAC TGA ATG AC-3′) and reverse (5′-GCC TTG GAA CAT AGT CTG AC-3′). The housekeeping gene glyceraldehyde-3-phosphate dehydrogenase (*GAPDH*) was used as the internal control, and the primers used were those described by Qi et al. (2017). The data were presented as the fold changes in relative gene expression normalized to the level of *GAPDH* expression and relative to the levels in the negative stimulation controls.

### 2.9 Conservation of the identified potential SLA Hp-4.0 haplotype-restricted CTL epitopes in M protein of genotype II PRRSV strains

To determine the sequence conservation of the identified potential SLA Hp-4.0 haplotype-restricted CTL epitopes, the amino acid sequences of the M protein of 248 genotype II PRRSV strains collected between 1998 and 2019 were downloaded from GenBank (Supplementary Table S2), including 236 strains from China, 7 strains from the United States, and 5 strains from other countries. Multiple sequence alignments were constructed using Geneious Basic software (Kearse et al., 2012).

### 2.10 Statistical analysis

All data were presented as means ± standard deviations. One-way analysis of variance was used for multigroup comparisons, and Student’s *t*-test was used for pairwise comparisons. Statistical analyses and data plotting were performed with GraphPad Prism (version 5.0) software (GraphPad Software Inc., La Jolla, CA). All comparisons were considered statistically significant at *p* < 0.05 and highly significant at *p* < 0.01.

## 3 Results

### 3.1 Specific binding of predicted epitope peptides to SLA-1*04:01:01 molecule

**Figure 1:**
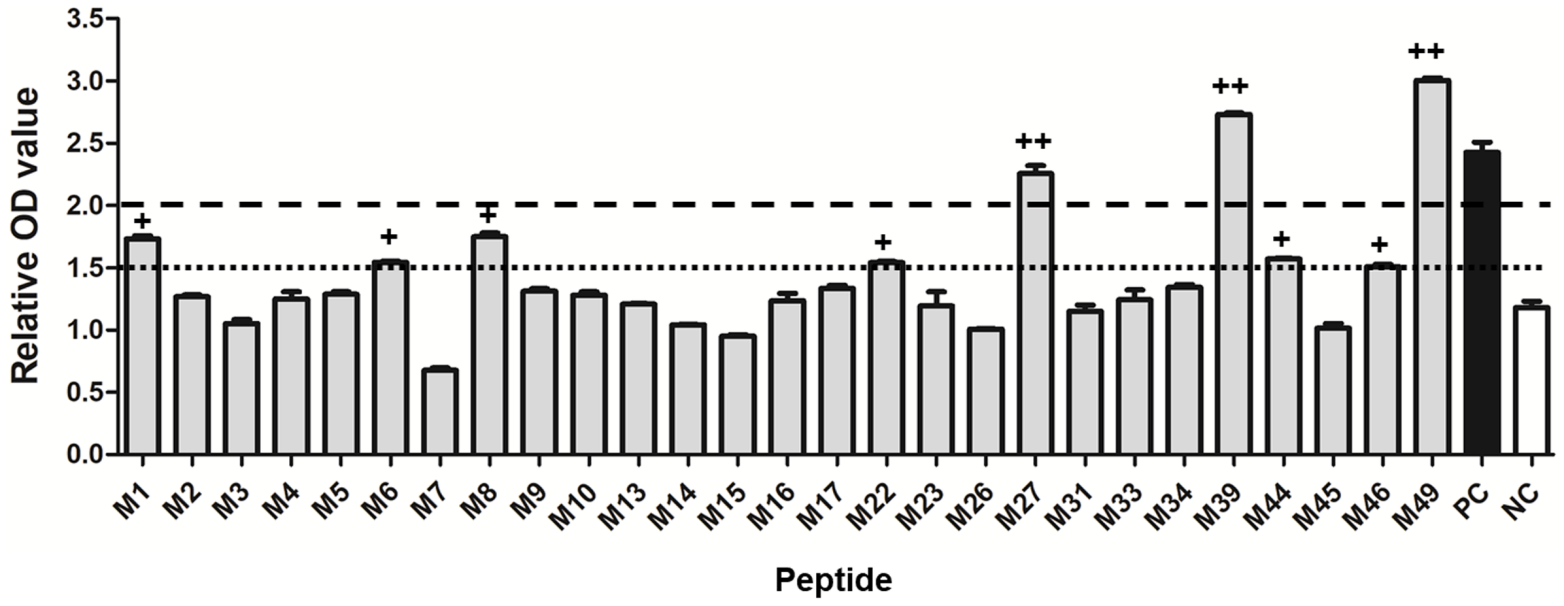
Specific binding of the predicted 9-mer epitope peptides of PRRSV M protein to SLA-1*04:01:01 molecule. “++” indicates that the epitope peptide specifically binds to the SLA-1*04:01:01 molecule (relative OD value ≥2). “+” indicates a lower capacity of the epitope peptide to bind SLA-1*04:01:01 (1.5 < relative OD values < 2.0). PC: positive control; NC: negative control. Each sample was assayed in triplicate to determine the mean value.

A total of 27 potential SLA-1*040101-restricted epitope peptides of the PRRSV M protein with high binding scores were predicted and synthesized. In total, 27 pET-epitope-SLA-1*040101-β2m complexes were constructed and expressed. All recombinant protein products were inducible with 1 mmol/L IPTG and were not produced in non-induced cultures. SDS-PAGE showed that the transformed cells produced a large amount of protein with a mass of approximately 44 kDa (Supplementary Figure S2). All the recombinant proteins were present as insoluble inclusion bodies. Effective inclusion of body purification and protein refolding produced activated proteins of adequate purity (Supplementary Figure S2). Subsequently, the specific binding of predicted epitope peptides to the SLA-1*04:01:01 molecule was determined with a previously reported ELISA-based method. The results indicated that M27, M39, and M49 were specifically bound to SLA-1*04:01:01 (relative OD values >2.0). Moreover, the lower capacities of M1, M6, M8, M22, M44, and M46 to bind SLA-1*04:01:01 were detected (1.5 < relative OD values <2.0) (Figure 1).

### 3.2 Specific binding of SLA-1*040101-binding peptides to SLA-2*04:01 and SLA-3*04:01 molecules

**Figure 2:**
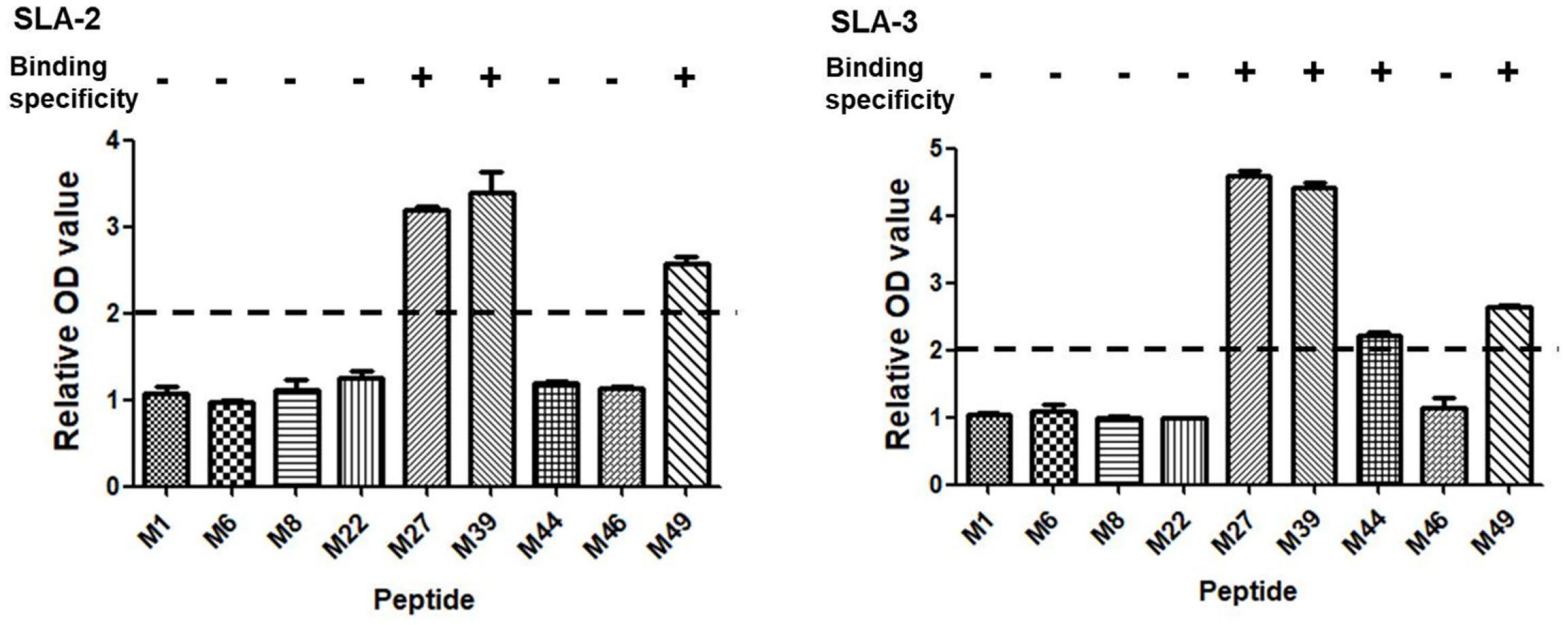
Specific binding of the screened SLA-1*04:01:01-binding epitope peptides of PRRSV M protein to SLA-2*04:01 and SLA-3*04:01 molecules. “+” indicates that the epitope peptide specifically binds to SLA-2 and SLA-3 molecules (relative OD value ≥ 2), respectively. Each sample was assayed in triplicate to determine the mean value.

Based on the screening results of the predicted epitope peptides to bind SLA-1*04:01:01, nine pET-epitope-SLA-2*0401-β2m complexes and nine pET-epitope-SLA-3*0401-β2m complexes were constructed (“epitope” indicates M1, M6, M8, M22, M27, M39, M44, M46, or M49), and their specificity of binding was analyzed. Similar situations revealed confirmations that M27, M39, and M49 bind specifically to SLA-2*04:01 and SLA-3*04:01, respectively (Figure 2).

### 3.3 Proliferation of PBMCs isolated from the vaccinated piglets induced by predicted epitope peptides

The SLA class I genotypes of each Landrace piglet were determined according to the nucleotide sequences of *SLA-1*, *SLA-3*, and *SLA-2* alleles. The SLA Hp-4.0 piglets carrying homozygous SLA-1*04:01:01, SLA-3*04:01, and SLA-2*04:01 alleles and the SLA Hp-26.0 piglets carrying homozygous SLA-1*08:11, SLA-3*05:02, and SLA-2*10:06 alleles were selected for the vaccination experiment. The specific anti-PRRSV antibodies in the serum samples from all vaccinated piglets were analyzed with an ELISA. All the piglets were seronegative for anti-PRRSV antibodies before vaccination. After vaccination, the antibody responses of three Hp-4.0 piglets and three Hp-26.0 piglets indicated that they had seroconverted on DPV 7. The antibodies increased gradually in the Hp-26.0 piglets till the end of the experiment. In the Hp-4.0 piglets, the antibody levels increased gently after DPV 14 (Supplementary Figure S3). No anti-PRRSV antibodies were detected in the negative control pigs during the experimental period.

**Figure 3:**
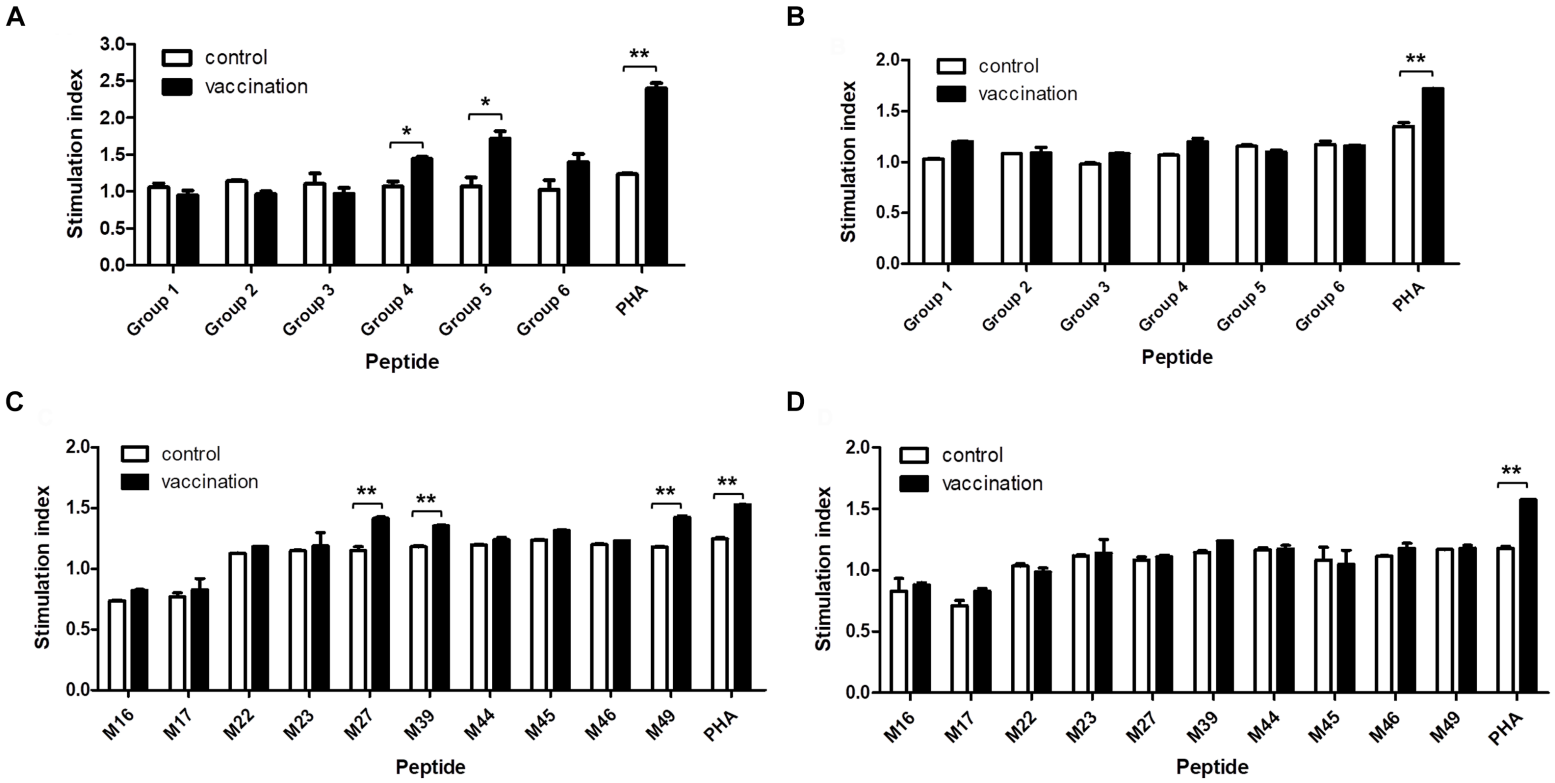
Proliferation of PBMCs induced by the predicted epitope peptide groups and the individual peptide in selected groups. PBMCs were prepared from PRRSV HuN4-F112-vaccinated Hp-4.0 and Hp-26.0 haplotype piglets and unvaccinated piglets at 17 days post vaccination (DPV). PBMCs from Hp-4.0 **(A)** and Hp-26.0 haplotype piglets **(B)** and unvaccinated piglets were stimulated with six peptide groups for 72 h. PBMCs from Hp-4.0 **(C)** and Hp-26.0 haplotype piglets **(D)** and unvaccinated piglets were stimulated with 10 epitope peptides from groups 4 and 5 for 72 h. PHA was used as the positive control and ddH_2_O or DMSO was used as the negative control. The PBMC proliferation response was determined and expressed as the stimulation index, which was calculated as the ratio of the average OD of peptide-stimulated cells to the average OD of unstimulated cells for each SLA haplotype. *p < 0.05; **p < 0.01.

The proliferation of PBMCs isolated from the homozygous Hp-4.0 and Hp-26.0 haplotype piglets induced with the predicted epitope peptides was tested to evaluate the immunological activities of the peptides. In the first round of screening, after stimulation with peptide group 4 or group 5, the stimulation index in the vaccinated group of the Hp-4.0 haplotype was significantly higher than that in the control group (Figure 3A). For the Hp-26.0 haplotype, there was no significant difference in the stimulation index in the vaccinated group compared with the control group after stimulation with any peptide groups (Figure 3B). Based on the results of the first screening, 10 predicted peptides in groups 4 and 5 were screened individually for their ability to stimulate PBMCs. Peptides M27, M39, and M49 efficiently induced the PBMCs isolated from the vaccinated Hp-4.0 haplotype piglets to proliferate, and the stimulation index was significantly higher than those of the control group (Figure 3C). However, no obvious proliferation of PBMCs isolated from the vaccinated Hp-26.0 haplotype piglets was observed in response to stimulation with 10 peptides (Figure 3D).

### 3.4 Verification of the immunogenic CTL epitopes with ELISpot assay

**Figure 4:**
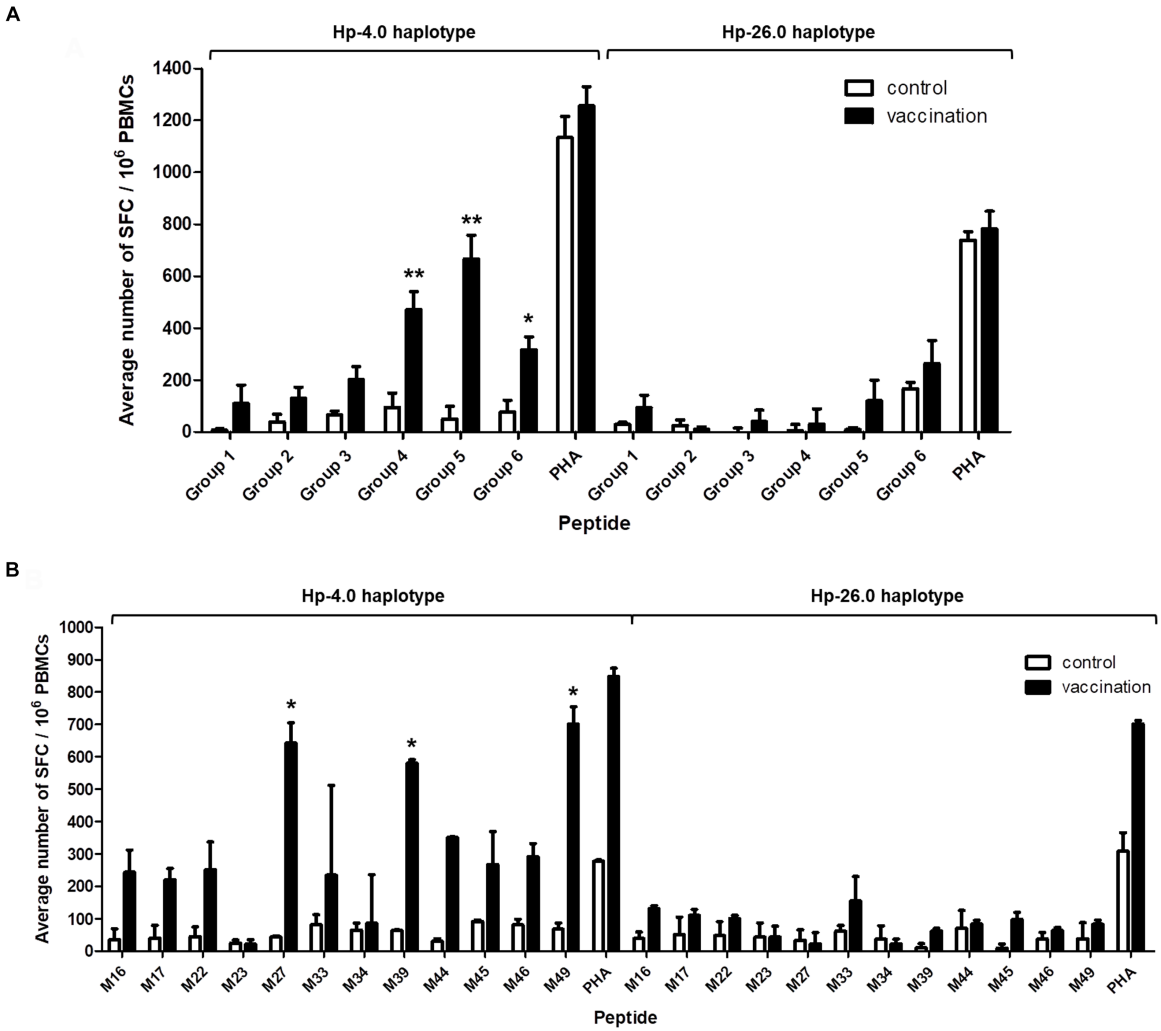
Detection of IFN-γ secretion from PBMCs induced with the predicted epitope peptide groups and the individual peptide in selected groups with an ELISpot assay. PBMCs isolated from PRRSV HuN4-F112-vaccinated Hp-4.0 or Hp-26.0 haplotype piglets and unvaccinated piglets were seeded in 96-well plates pre-coated with anti-IFN-γ antibody. After stimulation with six groups of peptides, the spot forming cells (SFC) were counted. PHA was used as the positive control and ddH_2_O or DMSO was used as the negative control. The results are expressed as the average number of SFC per one million PBMCs (SFC/10^6^ PBMCs). The predicted epitope peptide groups **(A)** and 12 predicted peptides in groups 4, 5, and 6 **(B)** were used for the IFN-γ ELISpot assay, respectively. *p < 0.05; **p < 0.01.

An ELISpot assay was used to determine whether the predicted CTL epitopes stimulated IFN-γ secretion when restricted to a specific SLA background. The SLA Hp-4.0 haplotype-restricted T-cell immunodominant epitopes of the PRRSV M protein were confirmed based on the average number of SFC/10^6^ PBMCs. PBMCs from the vaccinated Hp-4.0 haplotype piglets were used to test their reactivity to each peptide group. The results showed that the IFN-γ secreting cells stimulated by peptide groups 4, 5, and 6 were significantly higher than those of peptide groups 1, 2, and 3. These results indicate that in each peptide group, there was at least one Hp-4.0 haplotype-restricted epitope that was recognized by PBMCs from the Hp-4.0 haplotype piglets and induced the PBMCs to secrete IFN-γ. The average numbers of SFC did not differ significantly among all the peptide groups when used to stimulate PBMCs from the vaccinated Hp-26.0 haplotype piglets (Figure 4A).

To identify the specific epitopes that play the key roles in activating PBMCs, peptides M16, M17, M22, M23, M27, M33, M34, M39, M44, M45, M46, and M49 were tested. Peptides M27, M39, and M49 elicited the highest expression levels of IFN-γ expression, and the average number of stimulated PBMCs from the vaccinated Hp-4.0 haplotype piglets was significantly higher after stimulation with M27, M39, or M49 compared to stimulation with the other peptides. In response to stimulation with the M33 peptide from group 6, a large fluctuation in IFN-γ secretion was observed for PBMCs from the vaccinated Hp-4.0 piglets. Therefore, the M33 peptide was excluded from further analysis. The vaccinated Hp-26.0 haplotype group showed no significant changes in the average number of IFN-γ SFC when PBMCs were stimulated with the 12 peptides (Figure 4B; Supplementary Figure S4). Therefore, peptides M27, M39, and M49 were identified as the optimal potential SLA Hp-4.0 haplotype-restricted T-cell immunodominant epitope peptides.

### 3.5 Analysis of IFN-γ expression at the transcriptional level

**Figure 5:**
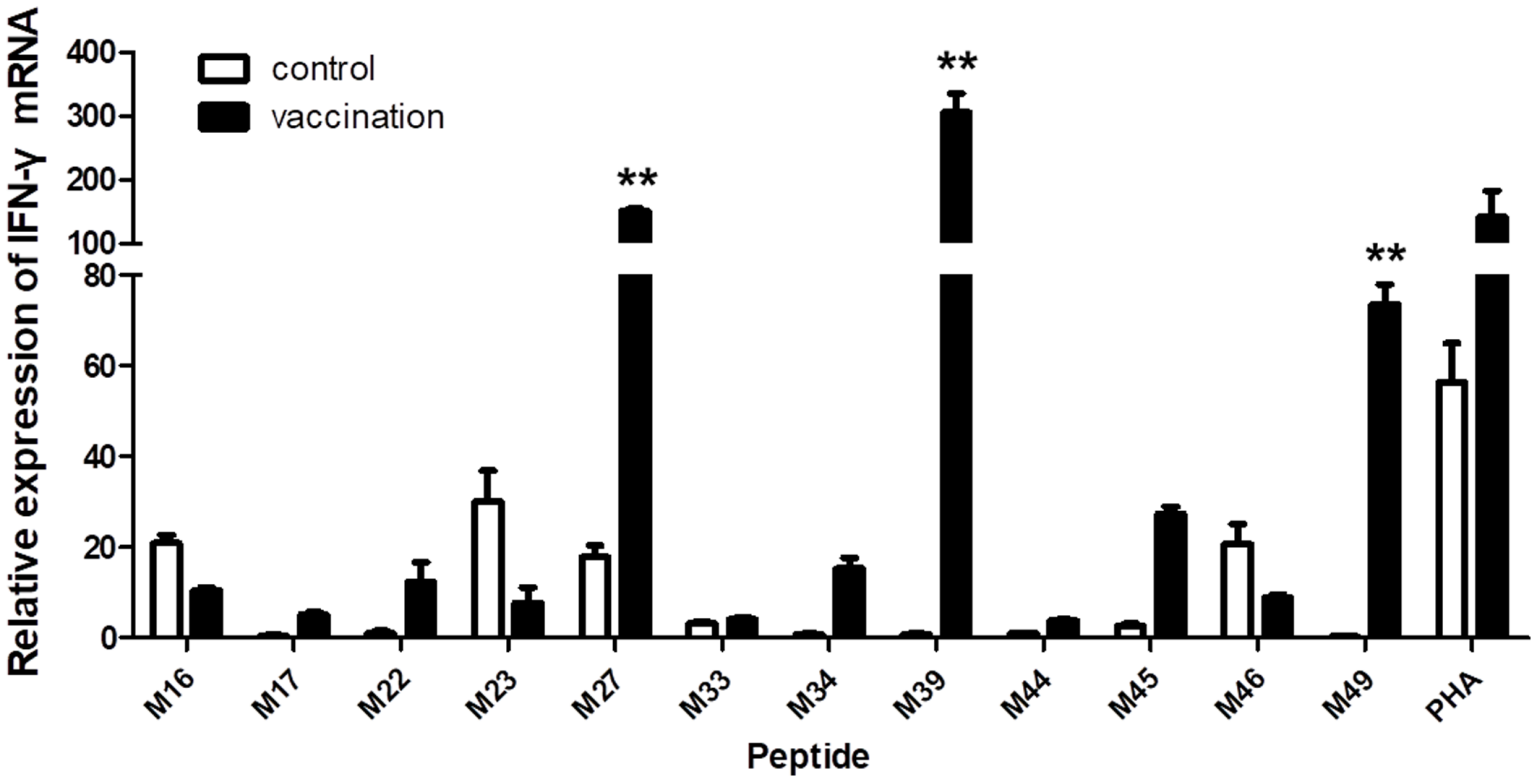
Changes in IFN-γ transcript expression induced by stimulation with individual predicted epitope peptides in groups 4, 5, and 6. PBMCs were isolated from PRRSV HuN4-F112-vaccinated or unvaccinated Hp-4.0 haplotype piglets. IFN-γ mRNA expression was determined with real-time qPCR. **p < 0.01.

When the PBMCs from the vaccinated Hp-4.0 haplotype piglets were co-cultured with individual peptides from groups 4, 5, and 6, the relative expression level of IFN-γ mRNA by PBMCs stimulated with M27, M39, or M49 peptides was highly significantly elevated compared with that in cells stimulated with the other peptides (Figure 5), indicating that these three peptides induced greater transcription of IFN-γ in SLA Hp-4.0 haplotype PBMCs.

### 3.6 Conservation of identified potential SLA Hp-4.0 haplotype-restricted CTL epitopes in M protein of genotype II PRRSV strains

**Figure 6:**
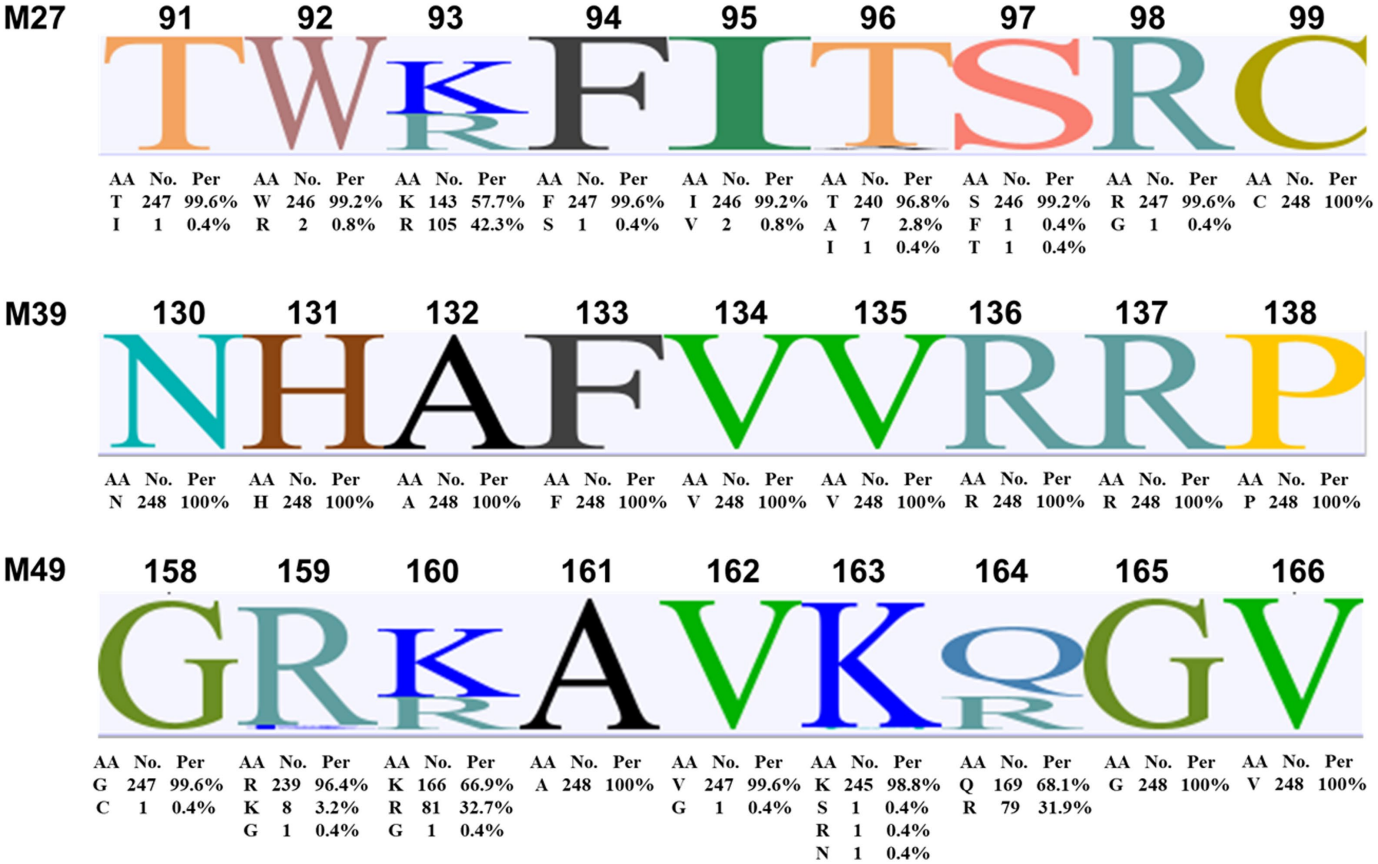
Amino acid sequence alignment of the identified potential SLA Hp-4.0 haplotype-restricted CTL epitopes M27, M39, and M49 in M proteins of genotype II PRRSV strains. The M protein amino acid sequences of 248 genotype II PRRSV strains collected between 1998 and 2019 were aligned with Geneious Basic software. AA, amino acid; No., number; Per, percent.

The amino acid sequences of the identified potential SLA Hp-4.0 haplotype-restricted CTL epitopes of the M protein of 248 genotype II PRRSV strains were aligned. The amino acid sequence of epitope M39 (NHAFVVRRP) was universally conserved in all 248 genotype II PRRSV strains. The amino acid sequences corresponding to epitope M27 (TWKFITSRC) were highly homologous to those of these PRRSV strains. Of the 248 sequences examined, 109 were identical to and completely consistent with the synthesized epitope peptide M27, whereas amino acid substitutions were found in the other strains, including T91I, W92R, K93R, F94S, I95V, T96A/I, S97F/T, and R98G. The main amino acid replacement occurred at position 93 (57.7% K and 42.3% R). Similar to M27, an amino acid sequence consistent with epitope M49 (GRKAVKQGV) was identified in 94 PRRSV strains. The amino acid substitutions in the other strains included G158C, R159K/G, K160R/G, V162G, K163S/R/N, and Q164R. Two amino acids in the M49 epitope, K (66.9%) and Q (68.1%), at positions 160 and 164, respectively, were replaced with high frequency in the other strains by amino acids R/G (32.7%/0.4%) and R (31.9%), respectively (Figure 6).

## 4 Discussion

PRRS has become an important disease in the Chinese pig industry since it was first reported in China in 1996 (Firth et al., 2011). With the continuous and rapid variation of PRRSV genes and antigens, current vaccines against PRRSV are not entirely satisfactory. PRRS has been one of the most challenging research topics in veterinary viral immunology. Previous studies have shown that cell-mediated immunity plays an important role in clearing PRRSV (Mateu and Diaz, 2008). A key effector cell in this task is the CTL, which can kill specific target cells directly and also induces the host cell-mediated specific immune response by simultaneously identifying viral epitope peptides specifically bound to SLA class I molecules and SLA class I molecules (SLA restriction). Therefore, SLA class I haplotype-restricted CTL epitopes are required to induce optimal anti-PRRSV immunity for the control and treatment of PRRSV infection. According to previous studies, the M polypeptide of PRRSV plays a major role in stimulating cellular immunity, and the M protein has been considered for the development of an efficacious vaccine (Jeong et al., 2010). The M protein is also a structural protein highly conserved among diverse PRRSV strains (Kappes and Faaberg, 2015). The T-cell responses induced by the epitope peptides derived from non-structural proteins appear to be less robust than those induced by epitope peptides from structural proteins (Cao et al., 2019). Therefore, it is essential to identify the SLA class I haplotype-restricted CTL epitopes in the M protein of PRRSV because they are important in monitoring CD8^+^ T-cell immune responses and in the development of a T-cell-based vaccine.

For a vaccine to induce a cell-mediated immune response, more specifically, the antigen must be presented on an SLA class I molecule as a 9-mer epitope for subsequent recognition by a cognate CTL. Therefore, an ensemble of SLA class I haplotype-restricted CTL epitopes that can provide protection to a population of diverse haplotypes must consist of epitopes that will bind collectively to the majority of SLA haplotypes present in the pig population. Although different pig populations have a rich diversity of SLA haplotypes (Hammer et al., 2020), certain alleles and the corresponding haplotypes are identified more frequently in swine breeds worldwide. The Hp-4.0 haplotype (SLA-1*04:01:01-SLA-3*04:01-SLA-2*04:01) is one of the most prevalent class I haplotypes in different pig breeds and swine cell lines, including Yorkshire, Landrace, NIH, Duroc, Meishan, Yucatan, Danish pigs, German purebred Landrace, outbred pig populations, and the PK13 and PK15 cell lines (Oleksiewicz et al., 2002; Smith et al., 2005; Ho et al., 2006, 2009a,b; Pedersen et al., 2014; Choi et al., 2016; Gimsa et al., 2017; Gao et al., 2017b; Techakriengkrai et al., 2021), indicating that Hp-4.0 is a valuable SLA class I haplotype that has survived long-term evolutionary selection. Therefore, it has wide application value in identifying SLA Hp-4.0 haplotype-restricted CTL epitopes for the rational design of veterinary vaccines.

Several studies have attempted to identify potential T-cell epitopes of the M protein of PRRSV using various methods, including intracellular cytokine staining, ELISpot assay, affinity and stability assays, and flow cytometry (Wang et al., 2011; Zhang et al., 2011; Welner et al., 2017; Cao et al., 2019). However, the SLA restriction of some of these identified peptides is unknown. The immunological activity of some epitope peptides has not been verified in pigs. More accurately, the crystal structure of the peptide-SLA-1*1502 complex was solved to illuminate the SLA-1*1502-restricted epitopes of PRRSV based on the peptide-binding motif, and then, the CTL immunogenicity of the identified epitopes was evaluated with SPF swine expressing SLA-1*1502 alleles (Pan et al., 2020). Unfortunately, no SLA-1*1502-restricted epitopes of M protein were identified. Furthermore, three 9-mer CTL epitopes of the M protein and the SLA molecules (SLA-2*0502 and SLA-2*1201) were identified, and these epitopes were confirmed with ELISpot to efficiently stimulate PBMCs with specific SLA genetic backgrounds to secrete IFN-γ (Liang et al., 2021). However, a major drawback of these studies was that IFN-γ secretion was measured in PBMCs from pigs with known *SLA-1* or *SLA-2* alleles but with little information on the SLA haplotype diversity. Given the strong linkage disequilibrium exhibited by the SLA loci, it is sometimes more appropriate and convenient for researchers to communicate and present findings in terms of haplotypes than in terms of individual allele specificities. Increasing evidence also suggests that the number of class I loci expressed is haplotype-specific (Lunney et al., 2009). Therefore, it is more important to identify SLA haplotype-restricted epitopes than SLA class I allele restriction, especially for homozygous SLA haplotypes.

In this study, we identified three potential SLA Hp-4.0 haplotype-restricted CTL epitopes in the M protein of PRRSV. First, 27 *in silico* predicted peptides with higher binding scores for SLA-1*04:01:01 were synthesized to construct the corresponding peptide-SLA-β2m complexes *in vitro*. The specific binding of these peptides to SLA-1*04:01:01, SLA-2*04:01, and SLA-3*04:01 was analyzed with an ELISA-based method to define the SLA restriction. Next, the 27 T-cell immunodominant epitopes were evaluated with CCK8, ELISpot, and real-time qPCR methods. The results showed that peptides M27, M39, and M49 induced significant proliferation and IFN-γ secretion in PBMCs from SLA Hp-4.0 haplotype piglets compared with those from Hp-26.0 haplotype piglets, which had been vaccinated with the commercial PRRSV HuN4-F112 strain. Meanwhile, we developed a more comprehensive screening method for SLA haplotype-restricted CTL epitopes. Because many methods were used in combination, our strategy was more accurate than individual methods. Although *in silico* prediction and the detection of the specific binding of epitope peptides to SLA molecules *in vitro* improve the efficiency of screening for SLA-restricted peptides, these methods have some limitations. Whether the epitopes identified actually play a biological role *in vivo* is unclear, and it must be finally verified in the host animal. In our study, although the low binding capacity of peptides M1, M6, M8, M22, M44, and M46 to SLA-1*04:01:01 *in vitro* was shown, they did not efficiently induce either the proliferation of or the secretion of IFN-γ by PMBCs from Hp-4.0 haplotype piglets. Furthermore, it is also essential to screen homozygous SLA haplotype piglets to confirm the immunological activity of the selected epitope peptides and to exclude the interference of other SLA class I alleles.

Additionally, identifying conserved epitopes not only ensures the highest degree of pathogen coverage achieved with the smallest number of epitopes but also excludes epitopes that are not essential to the pathogen. However, given the extensive heterogeneity, evolution, and recombination among PRRSV strains (Zhao et al., 2015; Gao J. C. et al., 2017; Liu et al., 2017), identifying a single immunogenic epitope expressed by all circulating strains of PRRSV is highly unlikely, and the CTL epitopes identified in PRRSV strains are usually not conserved. Fortunately, in this study, epitope M39 was universally conserved in all 248 genotype II PRRSV strains tested, and the epitopes represented by peptides M27 and M49 only showed one or two high-frequency amino acid replacements.

In conclusion, we have identified three potential SLA Hp-4.0 (SLA-1*04:01:01-SLA-3*04:01-SLA-2*04:01) haplotype-restricted CTL epitopes, namely, M27 (T_91_WKFITSRC), M39 (N_130_HAFVVRRP), and M49 (G_158_RKAVKQGV), in the M protein of PRRSV. These epitopes efficiently induced both the proliferation of PBMCs and the secretion of IFN-γ by PBMCs from piglets of the prevalent SLA Hp-4.0 haplotype vaccinated with the PRRSV strain. These three epitopes are also highly conserved among 248 genotype II PRRSV strains collected between 1998 and 2019. In this study, we developed an integrated approach that can be applied to the identification of SLA class I-restricted CTL epitopes of various important viruses, which will be useful in designing effective broad-spectrum peptide-based vaccines for swine.

## Data availability statement

The original contributions presented in the study are included in the article/Supplementary material, further inquiries can be directed to the corresponding authors.

## Ethics statement

The animal study was approved by the Animal Ethics Committee of HVRI, CAAS, China. The Animal Ethics Committee approval number is Heilongjiang Province, China no. 180428-01. The study was conducted in accordance with the local legislation and institutional requirements.

## Author contributions

TL: Writing – original draft. CXin: Writing – original draft. HL: Writing – original draft. CL: Writing – original draft. HC: Writing – original draft. CXia: Writing – review & editing. CG: Writing – review & editing.

## Footnotes

^1^ https://www.ebi.ac.uk/ipd/mhc/group/SLA

^2^ http://sb.nhri.org.tw/ICES

^3^ http://tools.immuneepitope.org/main/

^4^ http://www.cbs.dtu.dk/services/NetMHCpan/

## Supplementary material

SUPPLEMENTARY FIGURE S1 Construction of the pET-epitope-SLA-1*040101-β2m expression system. Horizontal boxes represent the amplified *SLA-1**04:01:01 allele and *β2m* gene sequence, the glycine-rich linker sequence, the C-terminal His-tag sequence derived from the pET-30a(+) prokaryotic expression vector, and the predicted CTL epitope peptide sequence. Upper case letters outside boxes indicate amino acids. Bold letters indicate restriction enzyme sites. Arc represents the pET-30a(+) vector.

SUPPLEMENTARY FIGURE S2 Analysis of inclusion body proteins by SDS-PAGE. M: protein marker; lane 1: the recombinant protein before induction with IPTG; lane 2: the recombinant protein after induction with IPTG; lane 3: the dissolved recombinant protein; lane4: the refolded recombinant protein.

SUPPLEMENTARY FIGURE S3 Detection of specific anti-PRRSV antibodies with ELISA. Serum samples were collected from all Hp-4.0 and Hp-26.0 haplotype piglets at 0, 1, 7, 14, and 19 days post vaccination (DPV).

SUPPLEMENTARY FIGURE S4 The display diagram of the spots forming cells (SFC) per 5 × 10^5^ PBMCs induced with M49 peptide with an ELISpot assay. PBMCs isolated from PRRSV HuN4-F112-vaccinated Hp-4.0 or Hp-26.0 haplotype piglets and unvaccinated piglets were seeded in 96-well plates pre-coated with anti-IFN-γ antibody. After stimulation with M49 peptide, SFC was displayed. PHA was used as the positive control.
