# Supplementary figures and images for: Potential SLA Hp-4.0 haplotype-restricted CTL epitopes identified from the membrane protein of PRRSV induce cell immune responses
Source: Front Microbiol. 2024 May 22;15:1404558. doi: 10.3389/fmicb.2024.1404558 (PMC11150780; doi:10.3389/fmicb.2024.1404558)

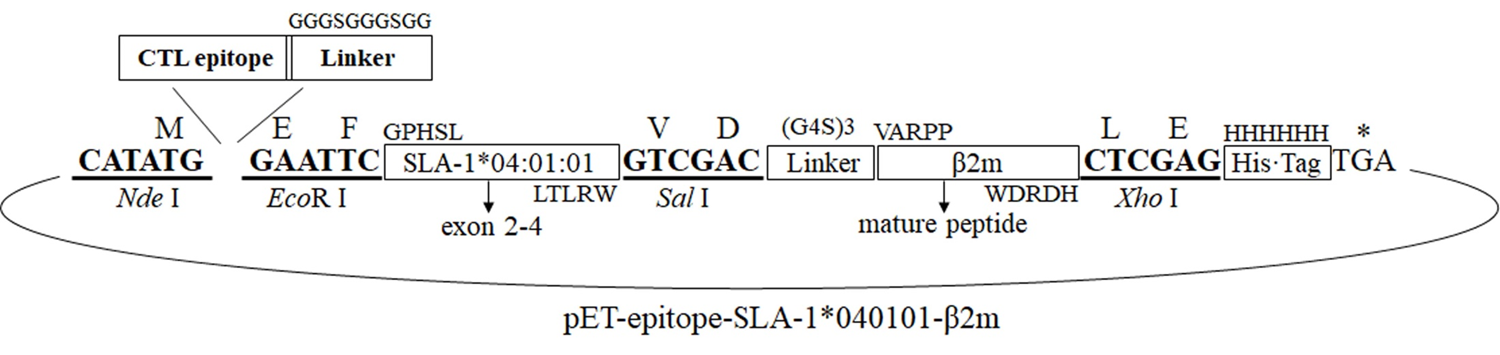

Supplement: SUPPLEMENTARY FIGURE S1 — Construction of the pET-epitope-SLA-1*040101-β2m expression system. Horizontal boxes represent the amplified SLA-1*04:01:01 allele and β2m gene sequence, the glycine-rich linker sequence, the C-terminal His-tag sequence derived from the pET-30a(+) prokaryotic expression vector, and the predicted CTL epitope peptide sequence. Upper case letters outside boxes indicate amino acids. Bold letters indicate restriction enzyme sites. Arc represents the pET-30a(+) vector. [file Image_1.TIF]

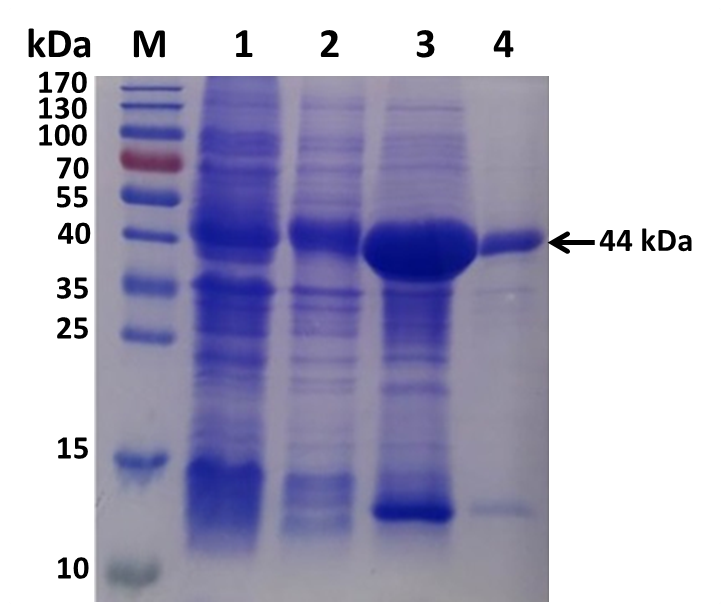

Supplement: SUPPLEMENTARY FIGURE S2 — Analysis of inclusion body proteins by SDS-PAGE. M: protein marker; lane 1: the recombinant protein before induction with IPTG; lane 2: the recombinant protein after induction with IPTG; lane 3: the dissolved recombinant protein; lane4: the refolded recombinant protein. [file Image_2.TIF]

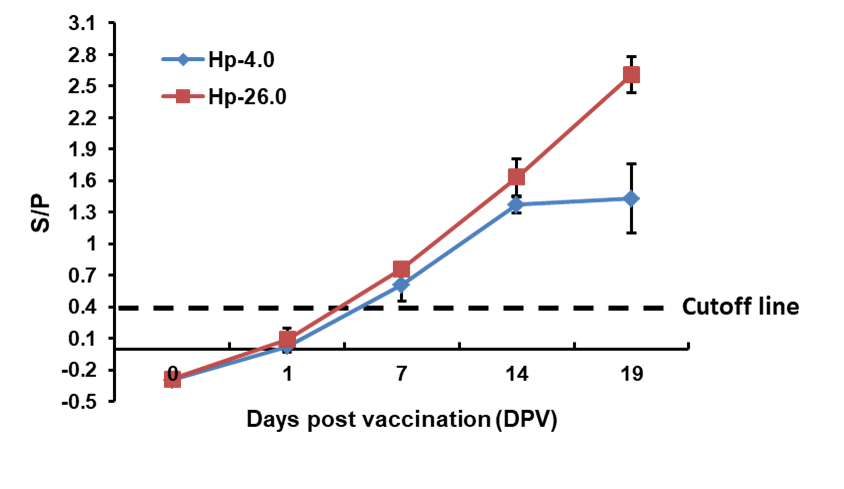

Supplement: SUPPLEMENTARY FIGURE S3 — Detection of specific anti-PRRSV antibodies with ELISA. Serum samples were collected from all Hp-4.0 and Hp-26.0 haplotype piglets at 0, 1, 7, 14, and 19 days post vaccination (DPV). [file Image_3.TIF]

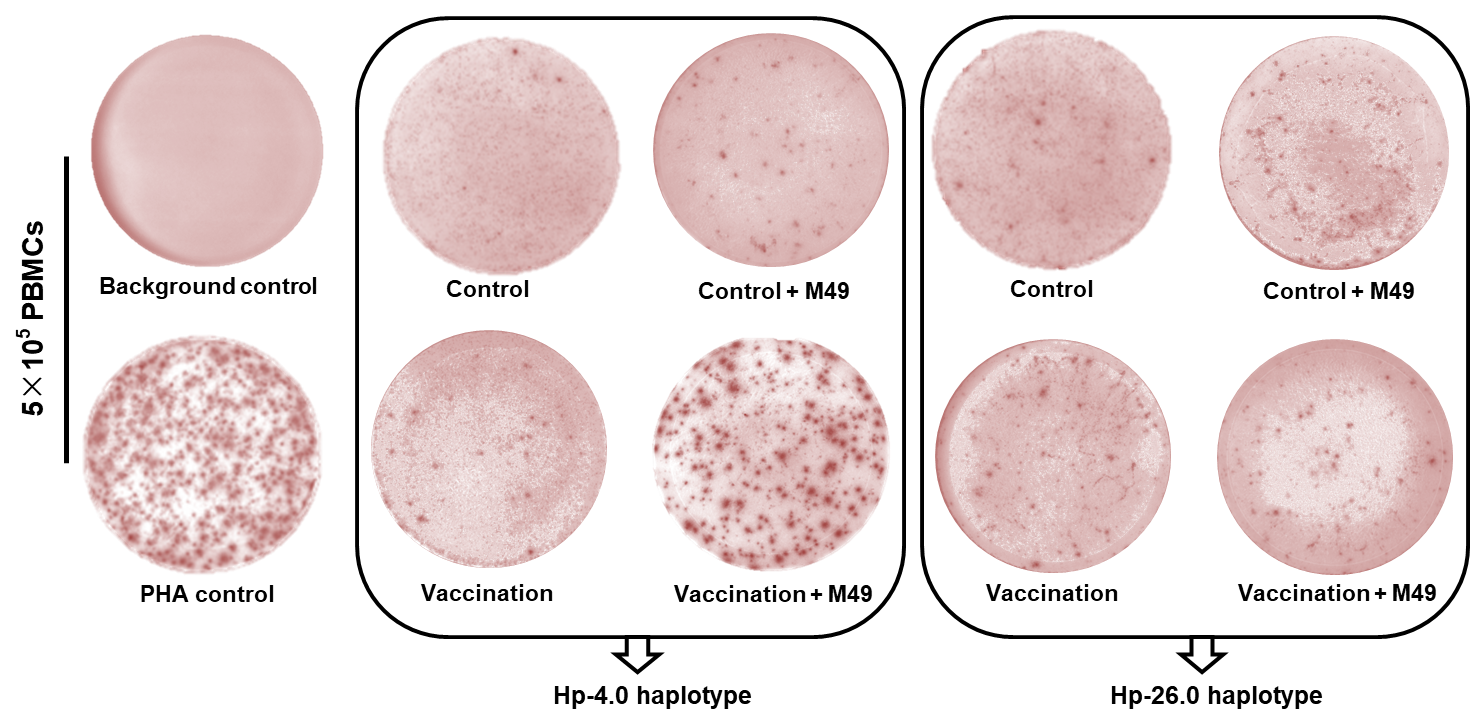

Supplement: SUPPLEMENTARY FIGURE S4 — The display diagram of the spots forming cells (SFC) per 5 × 10^5^ PBMCs induced with M49 peptide with an ELISpot assay. PBMCs isolated from PRRSV HuN4-F112-vaccinated Hp-4.0 or Hp-26.0 haplotype piglets and unvaccinated piglets were seeded in 96-well plates pre-coated with anti-IFN-γ antibody. After stimulation with M49 peptide, SFC was displayed. PHA was used as the positive control. [file Image_4.TIF]
