# Supplementary material for: Potential SLA Hp-4.0 haplotype-restricted CTL epitopes identified from the membrane protein of PRRSV induce cell immune responses
Source: Front Microbiol. 2024 May 22;15:1404558. doi: 10.3389/fmicb.2024.1404558 (PMC11150780; doi:10.3389/fmicb.2024.1404558)
Supplement: Supplementary file 5 [file Table_1.DOCX]

Supplementary Table S1 Nucleotides encoding predicted epitope peptides and the glycine-rich linker

| Name | Sequence (5′→3′) |
| --- | --- |
| M1 | S T A P Q K V L L  M-1F: **TATG** AGC ACA GCT CCA CAG AAG GTG CTT TTG GGTGGCGGTTCCGGCGGTGGCTCCGGCGGT **G** |
|  | M-1R: **AATTC** ACCGCCGGAGCCACCGCCGGAACCGCCACC CAAAAGCACCTTCTGTGGAGCTGTGCT **CA** |
| M2 | V L L A F S I T Y  M-2F: **TATG** GTG CTT TTG GCG TTT TCC ATT ACC TAC GGTGGCGGTTCCGGCGGTGGCTCCGGCGGT **G** |
|  | M-2R: **AATTC** ACCGCCGGAGCCACCGCCGGAACCGCCACC GTAGGTAATGGAAAACGCCAAAAGCAC **CA** |
| M3 | L K V S R G R L L  M-3F: **TATG** CTA AAG GTA AGT CGC GGC CGA CTG CTA GGTGGCGGTTCCGGCGGTGGCTCCGGCGGT **G** |
|  | M-3R: **AATTC** ACCGCCGGAGCCACCGCCGGAACCGCCACC TAGCAGTCGGCCGCGACTTACCTTTAG **CA** |
| M4 | G L L H L L I F L  M-4F: **TATG** GGG CTT CTG CAC CTT TTG ATC TTT CTG GGTGGCGGTTCCGGCGGTGGCTCCGGCGGT **G** |
|  | M-4R: **AATTC** ACCGCCGGAGCCACCGCCGGAACCGCCACC CAGAAAGATCAAAAGGTGCAGAAGCCC **CA** |
| M5 | F G Y M T F V H F  M-5F: **TATG** TTC GGG TAC ATG ACA TTC GTG CAC TTT GGTGGCGGTTCCGGCGGTGGCTCCGGCGGT **G** |
|  | M-5R: **AATTC** ACCGCCGGAGCCACCGCCGGAACCGCCACC AAAGTGCACGAATGTCATGTACCCGAA **CA** |
| M6 | H F E S T N R V A  M-6F: **TATG** CAC TTT GAG AGC ACA AAT AGG GTC GCG GGTGGCGGTTCCGGCGGTGGCTCCGGCGGT **G** |
|  | M-6R: **AATTC** ACCGCCGGAGCCACCGCCGGAACCGCCACC CGCGACCCTATTTGTGCTCTCAAAGTG **CA** |
| M7 | L T M G A V V A L  M-7F: **TATG** CTC ACT ATG GGA GCA GTA GTT GCA CTT GGTGGCGGTTCCGGCGGTGGCTCCGGCGGT **G** |
|  | M-7R: **AATTC** ACCGCCGGAGCCACCGCCGGAACCGCCACC AAGTGCAACTACTGCTCCCATAGTGAG **CA** |
| M8 | Y S A I E T W K F  M-8F: **TATG** TAC TCA GCC ATA GAA ACC TGG AAA TTC GGTGGCGGTTCCGGCGGTGGCTCCGGCGGT **G** |
|  | M-8R: **AATTC** ACCGCCGGAGCCACCGCCGGAACCGCCACC GAATTTCCAGGTTTCTATGGCTGAGTA **CA** |
| M9 | K F I T S R C R L  M-9F: **TATG** AAA TTC ATC ACC TCC AGA TGC CGT TTG GGTGGCGGTTCCGGCGGTGGCTCCGGCGGT **G** |
|  | M-9R: **AATTC** ACCGCCGGAGCCACCGCCGGAACCGCCACC CAAACGGCATCTGGAGGTGATGAATTT **CA** |
| M10 | T S R C R L C L L  M-10F: **TATG** ACC TCC AGA TGC CGT TTG TGC TTG CTA GGTGGCGGTTCCGGCGGTGGCTCCGGCGGT **G** |
|  | M-10R: **AATTC** ACCGCCGGAGCCACCGCCGGAACCGCCACC TAGCAAGCACAAACGGCATCTGGAGGT **CA** |
| M13 | D F C N D S T A P  M-13F: **TATG** GAC TTC TGC AAT GAT AGC ACA GCT CCA GGTGGCGGTTCCGGCGGTGGCTCCGGCGGT **G** |
|  | M-13R: **AATTC** ACCGCCGGAGCCACCGCCGGAACCGCCACC TGGAGCTGTGCTATCATTGCAGAAGTC **CA** |
| M14 | D S T A P Q K V L  M-14F: **TATG** GAT AGC ACA GCT CCA CAG AAG GTG CTT GGTGGCGGTTCCGGCGGTGGCTCCGGCGGT **G** |
|  | M-14R: **AATTC** ACCGCCGGAGCCACCGCCGGAACCGCCACC AAGCACCTTCTGTGGAGCTGTGCTATC **CA** |
| M15 | P Q K V L L A F S  M-15F: **TATG** CCA CAG AAG GTG CTT TTG GCG TTT TCC GGTGGCGGTTCCGGCGGTGGCTCCGGCGGT **G** |
|  | M-15R: **AATTC** ACCGCCGGAGCCACCGCCGGAACCGCCACC GGAAAACGCCAAAAGCACCTTCTGTGG **CA** |
| M16 | Q K V L L A F S I  M-16F: **TATG** CAG AAG GTG CTT TTG GCG TTT TCC ATT GGTGGCGGTTCCGGCGGTGGCTCCGGCGGT **G** |
|  | M-16F: **AATTC** ACCGCCGGAGCCACCGCCGGAACCGCCACC AATGGAAAACGCCAAAAGCACCTTCTG **CA** |
| M17 | V M I Y A L K V S  M-17F: **TATG** GTG ATG ATA TAT GCT CTA AAG GTA AGT GGTGGCGGTTCCGGCGGTGGCTCCGGCGGT **G** |
|  | M-17F: **AATTC** ACCGCCGGAGCCACCGCCGGAACCGCCACC ACTTACCTTTAGAGCATATATCATCAC **CA** |
| M22 | L H L L I F L N C  M-22F: **TATG** CTG CAC CTT TTG ATC TTT CTG AAT TGT GGTGGCGGTTCCGGCGGTGGCTCCGGCGGT **G** |
|  | M-22R: **AATTC** ACCGCCGGAGCCACCGCCGGAACCGCCACC ACAATTCAGAAAGATCAAAAGGTGCAG **CA** |
| M23 | T N R V A L T M G  M-23F: **TATG** ACA AAT AGG GTC GCG CTC ACT ATG GGA GGTGGCGGTTCCGGCGGTGGCTCCGGCGGT **G** |
|  | M-23R: **AATTC** ACCGCCGGAGCCACCGCCGGAACCGCCACC TCCCATAGTGAGCGCGACCCTATTTGT **CA** |
| M26 | I E T W K F I T S  M-26F: **TATG** ATA GAA ACC TGG AAA TTC ATC ACC TCC GGTGGCGGTTCCGGCGGTGGCTCCGGCGGT **G** |
|  | M-26R: **AATTC** ACCGCCGGAGCCACCGCCGGAACCGCCACC GGAGGTGATGAATTTCCAGGTTTCTAT **CA** |
| M27 | T W K F I T S R C  M-27F: **TATG** ACC TGG AAA TTC ATC ACC TCC AGA TGC GGTGGCGGTTCCGGCGGTGGCTCCGGCGGT **G** |
|  | M-27R: **AATTC** ACCGCCGGAGCCACCGCCGGAACCGCCACC GCATCTGGAGGTGATGAATTTCCAGGT **CA** |
| M31 | L G R K Y I L A P  M-31F: **TATG** CTA GGC CGC AAG TAC ATT CTG GCC CCT GGTGGCGGTTCCGGCGGTGGCTCCGGCGGT **G** |
|  | M-31R: **AATTC** ACCGCCGGAGCCACCGCCGGAACCGCCACC AGGGGCCAGAATGTACTTGCGGCCTAG **CA** |
| M33 | V E S A A G F H P  M-33F: **TATG** GTC GAA AGT GCC GCG GGC TTT CAT CCG GGTGGCGGTTCCGGCGGTGGCTCCGGCGGT **G** |
|  | M-33R: **AATTC** ACCGCCGGAGCCACCGCCGGAACCGCCACC CGGATGAAAGCCCGCGGCACTTTCGAC **CA** |
| M34 | G F H P I A A N D  M-34F: **TATG** GGC TTT CAT CCG ATT GCG GCA AAT GAT GGTGGCGGTTCCGGCGGTGGCTCCGGCGGT **G** |
|  | M-34R: **AATTC** ACCGCCGGAGCCACCGCCGGAACCGCCACC ATCATTTGCCGCAATCGGATGAAAGCC **CA** |
| M39 | N H A F V V R R P  M-39F: **TATG** AAC CAC GCA TTT GTC GTC CGG CGT CCC GGTGGCGGTTCCGGCGGTGGCTCCGGCGGT **G** |
|  | M-39R: **AATTC** ACCGCCGGAGCCACCGCCGGAACCGCCACC GGGACGCCGGACGACAAATGCGTGGTT **CA** |
| M44 | N G T L V P G L K  M-44F: **TATG** AAC GGC ACA TTG GTG CCC GGG TTG AAA GGTGGCGGTTCCGGCGGTGGCTCCGGCGGT **G** |
|  | M-44R: **AATTC** ACCGCCGGAGCCACCGCCGGAACCGCCACC TTTCAACCCGGGCACCAATGTGCCGTT **CA** |
| M45 | P G L K S L V L G  M-45F: **TATG** CCC GGG TTG AAA AGC CTC GTG TTG GGT GGTGGCGGTTCCGGCGGTGGCTCCGGCGGT **G** |
|  | M-45R: **AATTC** ACCGCCGGAGCCACCGCCGGAACCGCCACC ACCCAACACGAGGCTTTTCAACCCGGG **CA** |
| M46 | L K S L V L G G R  M-46F: **TATG** TTG AAA AGC CTC GTG TTG GGT GGC AGA GGTGGCGGTTCCGGCGGTGGCTCCGGCGGT **G** |
|  | M-46R: **AATTC** ACCGCCGGAGCCACCGCCGGAACCGCCACC TCTGCCACCCAACACGAGGCTTTTCAA **CA** |
| M49 | G R K A V K Q G V  M-49F: **TATG** GGC AGA AAA GCT GTT AAG CAG GGA GTG GGTGGCGGTTCCGGCGGTGGCTCCGGCGGT **G** |
|  | M-49R: **AATTC** ACCGCCGGAGCCACCGCCGGAACCGCCACC CACTCCCTGCTTAACAGCTTTTCTGCC **CA** |

Bold letters represent the sequences sticky to the ends of *Nde* I and *Eco*R I, respectively. Underlining indicates the glycine-rich linker sequence. The amino acid sequences of predicted epitope peptides are given above the forward sequences.
