# Supplementary material for: Potential SLA Hp-4.0 haplotype-restricted CTL epitopes identified from the membrane protein of PRRSV induce cell immune responses
Source: Front Microbiol. 2024 May 22;15:1404558. doi: 10.3389/fmicb.2024.1404558 (PMC11150780; doi:10.3389/fmicb.2024.1404558)
Supplement: Supplementary file 6 [file Table_2.DOCX]

Supplementary Table S2 Genotype II PRRSV strains and GenBank accession numbers used in this study

| Isolated strains | GenBank accession no. | Origin | Year of isolation |
| --- | --- | --- | --- |
| 16244B | AAC64689 | USA | 1998 |
| SP | AAF65939 | Singapore | 1999 |
| BJ-4 | AAG49625 | China | 2000 |
| ATCC VR-2332 | AAD12130 | USA | 2000 |
| CH-1a | AAK44217 | China | 2001 |
| HB-2(sh)/2002 | AAP57407 | China | 2002 |
| P129 | AAM18564 | USA | 2002 |
| PA8 | AAG13732 | Canada | 2002 |
| HN1 | AAR19406 | China | 2003 |
| Clone20 | ACP43268 | China | 2003 |
| NB/04 | ACL80325 | China | 2004 |
| NVSL 97-7895 | AAS59266 | USA | 2004 |
| PL97-1 | AAT00229 | South Korea | 2004 |
| 01NP1.2 | AAY53877 | Thailand | 2005 |
| RespPRRS vaccine | AAD27657 | USA | 2005 |
| MN184A | ABB18263 | USA | 2005 |
| GZ106 | AHZ89314 | China | 2005 |
| SHB | ACF94582 | China | 2005 |
| CC-1 | ABL98052 | China | 2006 |
| HUB1 | ABK63491 | China | 2006 |
| HUB2 | ABL60911 | China | 2006 |
| LMY | ABF19772 | South Korea | 2006 |
| Ingelvac ATP | ABJ51874 | USA | 2006 |
| rJXwn06 | ATU47071 | China | 2006 |
| JXwn06-81c | ADV78533 | China | 2006 |
| HN-HW | ACX33144 | China | 2006 |
| JSyx | ACH86031 | China | 2006 |
| TP | ACF94590 | China | 2006 |
| TJ | ACF76550 | China | 2006 |
| JX143 | ACD61701 | China | 2006 |
| HEB1 | ABL60919 | China | 2006 |
| JXA1 | ABL60903 | China | 2006 |
| S1 | ABE73143 | China | 2006 |
| 07HEN | ACJ12415 | China | 2007 |
| 07NM | ACJ12407 | China | 2007 |
| 07QN | ACJ06727 | China | 2007 |
| HB-1/3.9 | ABY66115 | China | 2007 |
| SHH | ABU94735 | China | 2007 |
| HV | AFS30910 | China | 2007 |
| DY | AFB82659 | China | 2007 |
| GDQY1 | AEW11429 | China | 2007 |
| Shaanxi-2 | ADY17434 | China | 2007 |
| BJSY07 | ADG45029 | China | 2007 |
| AH0701 | ADD10728 | China | 2007 |
| GDQY2 | ADC55003 | China | 2007 |
| 07BJ | ACJ12431 | China | 2007 |
| CG | ACF94574 | China | 2007 |
| NM1 | ACF76558 | China | 2007 |
| BJ | ACF49373 | China | 2007 |
| XH-GD | ACD03125 | China | 2007 |
| Henan-1 | ABW91087 | China | 2007 |
| Jiangxi-3 | ABW91079 | China | 2007 |
| WUH1 | ABW39368 | China | 2007 |
| SY0608 | ABV60272 | China | 2007 |
| GD | ABV02058 | China | 2007 |
| LN | ABV02050 | China | 2007 |
| NX06 | ABU55016 | China | 2007 |
| JXwn06 | ABR37303 | China | 2007 |
| HUN4 | ABR26254 | China | 2007 |
| HuN | ABS57408 | China | 2007 |
| SD-CXA/2008 | ACU31106 | China | 2008 |
| NT0801 P10 | AHZ34198 | China | 2008 |
| NT0801 | ADY86745 | China | 2008 |
| WUH3 | ADO33727 | China | 2008 |
| KP | ADA85033 | China | 2008 |
| 08HuN | ACZ54924 | China | 2008 |
| 08SDWF | ACZ57980 | China | 2008 |
| CWZ-1-F3 | ACQ71973 | China | 2008 |
| CBB-1-F3 | ACQ71966 | China | 2008 |
| PRRSV03 | ACH87577 | China | 2008 |
| PRRSV02 | ACH87569 | China | 2008 |
| PRRSV01 | ACH87561 | China | 2008 |
| CH-1R | ACF93753 | China | 2008 |
| SCwhn09CD | AFQ60937 | China | 2009 |
| ZCYZ | AEK75362 | China | 2009 |
| YD | AET07189 | China | 2009 |
| 09SC | AEL33533 | China | 2009 |
| HLJ-09 | ADZ28338 | China | 2009 |
| SY0909 | ADY86753 | China | 2009 |
| BB0907 | ADY86737 | China | 2009 |
| HLJHL | ADM64633 | China | 2009 |
| ZP-1 | ADG60170 | China | 2009 |
| 09HUB7 | ACZ57964 | China | 2009 |
| GD | ACZ67535 | China | 2009 |
| SD1-100 | ACY29604 | China | 2009 |
| SX-1 | ACW82443 | China | 2009 |
| JXM100 | ACV95345 | China | 2009 |
| GS2004 | ACG60148 | China | 2009 |
| GS2003 | ACG60140 | China | 2009 |
| GS2002 | ACG60132 | China | 2009 |
| WUH2 | ACZ58650 | China | 2009 |
| Shanxi-6 | AIS23655 | China | 2010 |
| GX1003 | AGC11951 | China | 2010 |
| JX | AFS30920 | China | 2010 |
| 10HN-GD | AGI50378 | China | 2010 |
| QY2010 | AFK65134 | China | 2010 |
| 10-10JX | AFK08993 | China | 2010 |
| FS | AEO92087 | China | 2010 |
| DC | AET07198 | China | 2010 |
| GX09-16 | ADK89212 | China | 2010 |
| YN-1 | AIE38326 | China | 2011 |
| WUH6 | ANT45970 | China | 2011 |
| GD-HD | AKU47855 | China | 2011 |
| GZ1101 | AHZ97946 | China | 2011 |
| LN1101 | AHW58238 | China | 2011 |
| BJ1102 | AHW58229 | China | 2011 |
| GD-2011 | AGJ51312 | China | 2011 |
| NJ-1106 | AFY23053 | China | 2011 |
| SDA2 | AGA16693 | China | 2011 |
| YN-2011 | AFV25450 | China | 2011 |
| HH08 | AFW90507 | China | 2011 |
| 11FS12-GD | AGI50477 | China | 2011 |
| NVDC-GD2-2011 | AFJ92657 | China | 2011 |
| WUH4 | AFG30133 | China | 2011 |
| GX10-48 | AFG25537 | China | 2011 |
| QYYZ | AFF61365 | China | 2011 |
| GM2 | AEZ01220 | China | 2011 |
| SD0901 | AEV46847 | China | 2011 |
| SC2012 | AKE49088 | China | 2012 |
| HENAN-HEB | AHN82317 | China | 2012 |
| XJu-1 | AHG97817 | China | 2012 |
| SH1211 | AHW29570 | China | 2012 |
| HZ-31 | AGH14483 | China | 2012 |
| SD16 | AFP25198 | China | 2012 |
| GX1001 | AFP47218 | China | 2012 |
| JL580 | AKZ66196 | China | 2013 |
| BJ-F20 | ALB08499 | China | 2013 |
| HEB 20130008-14 | ALD59984 | China | 2013 |
| MY-486 | AIC75895 | China | 2013 |
| HEB-2013 | AID16358 | China | 2013 |
| HeNan-A9 | AHZ96615 | China | 2013 |
| Henan-A4 | AHZ64899 | China | 2013 |
| HeNan-A1 | AHL83469 | China | 2013 |
| HENAN-XINX | AHB29376 | China | 2013 |
| HeNXX-2014-12 | QGY97965 | China | 2014 |
| Fujian-2014-18 | QGY97935 | China | 2014 |
| HeN1401 | AXF38006 | China | 2014 |
| GD1404 | AYE88569 | China | 2014 |
| GD1404 | ASK40172 | China | 2014 |
| FJM4 | ARV86041 | China | 2014 |
| FJL15 | ARV86034 | China | 2014 |
| HiNZWQ | ATU47347 | China | 2014 |
| JSWA | ATU47339 | China | 2014 |
| FJ1402 | APD14042 | China | 2014 |
| HENXX-1 | ANF99634 | China | 2014 |
| HNxa14 | ALU09374 | China | 2014 |
| CHsx1401 | ALB38656 | China | 2014 |
| 14LY02-FJ | AKU79230 | China | 2014 |
| NVDC-SD4-2014 | ALD60240 | China | 2014 |
| NVDC-SC1-2014 | ALD59880 | China | 2014 |
| TJbd14-1 | ALU09350 | China | 2014 |
| HUN-2014 | AKJ86985 | China | 2014 |
| BB0907-F44 | AIZ09148 | China | 2014 |
| HB2014001 | AIU95027 | China | 2014 |
| NMG2014 | AIS76363 | China | 2014 |
| Henan-A12 | AIJ19266 | China | 2014 |
| SC-d | AWK48879 | China | 2015 |
| SDhz1512 | ATH86653 | China | 2015 |
| HENJY-2 | ARN17982 | China | 2015 |
| 15ZJ1 | ARH13249 | China | 2015 |
| 15SC3 | ARH13217 | China | 2015 |
| 15LN3 | ARH13193 | China | 2015 |
| 15LN1 | ARH13177 | China | 2015 |
| 15JX1 | ARH13145 | China | 2015 |
| 15HEN4 | ARH13113 | China | 2015 |
| 15HEN1 | ARH13097 | China | 2015 |
| 15HEB1 | ARH13081 | China | 2015 |
| XJzx1-2015 | AQV08436 | China | 2015 |
| GDsg | ARG42314 | China | 2015 |
| GD-KP | ANS10128 | China | 2015 |
| HENZMD-9 | ANF99651 | China | 2015 |
| HENXC-4 | ANF99626 | China | 2015 |
| WUH5 | ANT45962 | China | 2015 |
| HNyc15 | ALU09398 | China | 2015 |
| JXja15 | ALU09366 | China | 2015 |
| FJFS | AKZ21444 | China | 2015 |
| FJE1 | AKZ21436 | China | 2015 |
| FJSD | AKZ21428 | China | 2015 |
| FJW05 | AKZ21402 | China | 2015 |
| FJY04 | AKZ21393 | China | 2015 |
| FJZ03 | AKZ21385 | China | 2015 |
| HLJ-80 | QGY97905 | China | 2016 |
| SD110-1608 | QIE30684 | China | 2016 |
| TJZH-1607 | AYO89562 | China | 2016 |
| SDQZ-1609 | AYO89542 | China | 2016 |
| SD99-1606 | AYO89532 | China | 2016 |
| SD53-1603 | AYO89522 | China | 2016 |
| SDQD-1604 | AYO89502 | China | 2016 |
| LNCH-1604 | AYO89492 | China | 2016 |
| HNJYH-1606 | AYO89482 | China | 2016 |
| HNJYF-1606 | AYO89462 | China | 2016 |
| HBFL-1604 | AYO89472 | China | 2016 |
| CY1-1604 | AYO89442 | China | 2016 |
| CY2-1604 | AYO89452 | China | 2016 |
| SDbz16-2 | QBO24030 | China | 2016 |
| ZJnb16-2 | QBA57443 | China | 2016 |
| GDZS2016 | AYH92594 | China | 2016 |
| HeN1601 | AXF38030 | China | 2016 |
| SCnj16 | ATG70528 | China | 2016 |
| SCcd16 | ATG70520 | China | 2016 |
| GDYDZZZ | AVA30169 | China | 2016 |
| SDYG1606 | ATW75090 | China | 2016 |
| HENXX-8 | ARN17990 | China | 2016 |
| SDlz1601 | ATH86662 | China | 2016 |
| HNhx | AOW71958 | China | 2016 |
| FJXS15 | ARA72294 | China | 2016 |
| FJWQ16 | ARA72284 | China | 2016 |
| SX2-1607 | QGY98095 | China | 2016 |
| SX1-1607 | QGY98085 | China | 2016 |
| HN-1603 | QGY98065 | China | 2016 |
| HEB-108 | QGY97925 | China | 2017 |
| SDWH27-1710 | QIE30673 | China | 2017 |
| GDsf1711 | QHA24438 | China | 2017 |
| GDsf1710 | QHA24430 | China | 2017 |
| GDsf1707 | QHA24422 | China | 2017 |
| GZgy17 | QCY50782 | China | 2017 |
| NADC30 | QBG05665 | China | 2017 |
| SCya17 | AXF35688 | China | 2017 |
| QHD3 | AYN07380 | China | 2017 |
| SCN17 | AXF35680 | China | 2017 |
| SD17-38 | AXF36026 | China | 2017 |
| SCcd17 | AWK22876 | China | 2017 |
| FJNP2017 | AYH92585 | China | 2017 |
| LNWK130 | AVM33070 | China | 2017 |
| LNWK96 | AVM33060 | China | 2017 |
| HB17A | AXS63576 | China | 2017 |
| QHD1 | AYJ76823 | China | 2017 |
| FJDJQ-2017 | AVW82208 | China | 2017 |
| FJLIUY-2017 | AVW82200 | China | 2017 |
| HeB-239 | QGY97975 | China | 2018 |
| GDsf1809 | QHA24486 | China | 2018 |
| CH-WH-2019-1 | QED58283 | China | 2018 |
| GDsf1808 | QHA24478 | China | 2018 |
| GDsf1807 | QHA24470 | China | 2018 |
| GDsf1806 | QHA24462 | China | 2018 |
| GDsf1804 | QHA24454 | China | 2018 |
| GDsf1802 | QHA24446 | China | 2018 |
| FJ0908 | QDA34023 | China | 2018 |
| SCya18 | QCY50790 | China | 2018 |
| GXNN1839 | QJI07989 | China | 2018 |
| HLJZD22-1812 | QIC50071 | China | 2018 |
| LNDZD10-1806 | QIC50037 | China | 2018 |
| JS18-3 | QJX74499 | China | 2018 |
| HLJ-DZD4-1805 | QGY98115 | China | 2018 |
| LN-DB87 | QGY98105 | China | 2018 |
| HLJWK108-1711 | QGY97985 | China | 2018 |
| HLHDZD32-1901 | QIC50061 | China | 2019 |
| HLJZD30-1902 | QIC50049 | China | 2019 |
